# Association between Genetic Variant of *Apolipoprotein C3* and Incident Hypertension Stratified by Obesity and Physical Activity in Korea

**DOI:** 10.3390/nu10111595

**Published:** 2018-10-30

**Authors:** Garam Jo, So-Young Kwak, Ji Young Kim, Hyunjung Lim, Min-Jeong Shin

**Affiliations:** 1 Department of Public Health Sciences, BK21PLUS Program in Embodiment: Health-Society Interaction, Graduate School, Korea University, Seoul 02841, Korea; grcho94@korea.ac.kr (G.J.); joilfille@korea.ac.kr (S.-Y.K.); kjy7598@hanmail.net (J.Y.K.); 2 Department of Medical Nutrition, Graduate School of East-West Medical Science, Kyung Hee University, Yongin 17104, Korea; hjlim@khu.ac.kr

**Keywords:** apolipoprotein C3, genetic variant, hypertension, obesity, physical activity, triglyceride

## Abstract

Apolipoprotein C3 (APOC3) is an important regulator of lipoprotein metabolism, and has been shown to be strongly associated with hypertriglyceridemia. We tested whether triglyceride-influencing genetic variants at *APOC3* (T-455C, C-482T, C1100T, and SstI) are associated with the onset of hypertension (HTN) among Korean adults stratified by lifestyle-related factors in the Ansung–Ansan cohort within the Korean Genome and Epidemiology Study. After excluding participants with preexisting cancer, cardiovascular diseases, diabetes, and HTN, a total of 5239 men and women were included at baseline (2001–2002), and followed up for a median of 9.8 years. Carriers of the C allele of C1100T with body mass index <25 kg/m^2^ showed a significantly lower HTN risk (hazard ratio (HR) than non-carriers: 0.87, 95% confidence interval (CI): 0.77–0.98) after adjusting for covariates. In addition, carriers of the C allele of T-455C and the T allele of C-482T with low physical activity had lower incident HTN than non-carriers (HR: 1.14, 95% CI: 1.03–1.26; HR: 1.13, 95% CI: 1.02–1.25). Our results suggest that genotype effects in *APOC3* on HTN risk have been shown in lean carriers of the C allele of C1100T and in less active people having the C allele of T-455C and T allele of C-482T in a large sample of the Korean population.

## 1. Introduction

Hypertension (HTN) is one of the factors contributing to the morbidity and mortality associated with cardiovascular disease (CVD) [1]. HTN prevalence has been continuously increasing, and the 2016 Korean National Health and Nutrition Survey reported that individuals having HTN accounted for 29.1% of the Korean adult population aged ≥30 years [2].

Hypertension is a complex disease with strong interaction between genetic susceptibility and environmental factors [3]. Among the risk factors affecting HTN incidence, several previous cross-sectional [4,5] and prospective observations [6,7] reported that lipid phenotypes characterized by increased triglyceride (TG) and decreased high-density-lipoprotein (HDL) cholesterol are associated with HTN risk. It has been speculated that disorders of lipoprotein metabolism might lead to endothelial dysfunction, altered nitric oxide production [8], structural changes in large arteries following atherosclerosis [9], and an overstimulated renin–angiotensin–aldosterone system induced by concomitant insulin resistance [10]. Apolipoprotein (apo) C3 is among the factors known to regulate plasma TG levels; it is a low molecular weight protein that exchanges among all lipoprotein particles, particularly very low-density lipoproteins (VLDL) and HDL. ApoC3 can inhibit the catabolism of TG-rich particles by both lipoprotein lipase and hepatic lipase, and can also impair the clearance of the lipolytic remnants of these particles from the bloodstream [11]. Moreover, apoCIII has been shown to have direct pro-inflammatory effects [12]. Collectively, these properties are felt to contribute to the risk for cardiovascular disease associated with increased plasma apoC3 concentrations [13]. The gene encoding apoC3 is on chromosome 11q23 within the *APOA1/C3/A4/A5* gene cluster [14]. Notably, rare loss-of-function mutations in *APOC3* are associated with both reduced plasma TG and decreased CVD risk [15]. A number of common single-nucleotide polymorphisms (SNPs) in the *APOC3* gene, including T-455C (rs2854116) and C-482T (rs2854117) in the promoter insulin-response element, C1100T (rs4520) in exon 3, and SstI (rs5128) in the 3′-UTR, have been extensively studied. The minor allele of SstI has shown to be in linkage disequilibrium with T-455C and C-482T [16]. These genetic variants have been associated in some reports with hypertriglyceridemia, low HDL cholesterol level, coronary heart disease, and/or nonalcoholic fatty liver disease [17,18,19]. On the other hand, lack of genetic associations of apoc3 variants with circulating TG, HDL cholesterol, and nonalcoholic fatty liver disease were also observed [20,21,22,23]. In addition to this, some studies have demonstrated that these mutations have an influence on *APOC3* concentration [24,25]. A previous study, conducted on Asian India men, has suggested that variants in the *APOC3* gene were related to increased serum *APOC3* levels [25]. Given the role of apoC3 in TG metabolism, and the association of dyslipidemias characterized by high TG and low HDL cholesterol with HTN, we tested whether common genetic variants of *APOC3* were associated with incident HTN according to the stratification of lifestyle-related factors, specifically, obesity and physical activity level, in a community-based Korean cohort.

## 2. Materials and Methods

### 2.1. Study Participants

The study population was the Ansan–Ansung cohort, which is the part of the Korean Genomic Epidemiologic Study (KoGES). A detailed description of the KoGES has been published elsewhere [26]. Briefly, the Ansan–Ansung cohort (KoGES-ASAS) is an ongoing prospective community-based cohort that was established in 2001–2002 to collect data from Koreans residing in urban (Ansan) and rural (Ansung) areas. For the baseline investigation, a total of 10,030 individuals aged 40–69 years were recruited in 2001–2002, and the participants were followed up biennially. Differences in baseline characteristics according to residence are as follows: Mean age of participants who lived in Ansan and Ansung was 47.5 and 53.6 years, respectively. Participants who resided in urban ares were more likely to be men, to have a higher body mass index (BMI), to do less exercise, to drink more and smoke less, and have a higher level of education and income than those who resided in rural areas. Also, the participants who were in urban areas developed HTN (20.9%) less than the participants in another area (36.1%) during 9.8 follow-up periods. However, the distributions of *APOC3* genotypes were similar in both area groups. Person-years for each participant were calculated from the date of administration of the baseline questionnaire to the date of the first HTN diagnosis, the date of last contact, or the end of the follow-up (November 2012), whichever came first. We excluded the loss of the follow-up period from the entirety of the person-years. The median follow-up period was 9.8 years. At each examination, data on demographic and lifestyle characteristics, metabolic profiles, medical history, and disease incidence were collected. For this study, 8841 subjects who completed DNA genotyping and quality control were investigated. Among them, participants with preexisting cancer (*n* = 104), CVD (*n* = 243), diabetes (*n* = 1060), and HTN (*n* = 2165) at the time of enrollment in the study were excluded. We also eliminated participants whose TG levels were >600 mg/dL (*n* = 30). Thus, the final group for analysis consisted of 5239 individuals. Informed consent was obtained from all study participants, and the study protocol was approved by the Institutional Review Board of the Korea Centers for Disease Control and Prevention (KBP-2016-062) and the Institutional Review Board at Korea University (KU-IRB-16-EX-272-A-1). The study was conducted in accordance with the Declaration of Helsinki.

### 2.2. General Characteristics

At each examination visit, participants in KoGES-ASAS were individually interviewed by trained technicians, and we obtained demographic and behavioral data, including age, gender, area, education level, physical activity, daily total energy intake, and smoking and drinking status, from questionnaire surveys. Education level was classified into four groups reflecting the highest educational level achieved by the participants: elementary school, middle school, high school, or university. Daily intakes of total energy were derived from semi-quantitative food frequency questionnaire, which was previously validated [27]. Smoking status and alcohol consumption were classified as current smokers/drinkers and nonsmokers/drinkers, and current smokers and current drinkers were defined as those who smoked cigarettes or drank alcoholic beverages at the time of the survey. Physical activity was assessed using the metabolic equivalents (METs, h/day). The value of total METs was calculated by multiplying the METs during each type of activity (2.4 for light, 5.0 for moderate, and 7.5 for intense activities) [28].

### 2.3. Anthropometric and Biochemical Measurements

For anthropometric and biochemical measurements, procedures and assay methods for KoGES-ASAS were used as described elsewhere [29]. Blood pressure (BP) measurements were measured twice to the nearest 2 mmHg with a mercury sphygmomanometer. Two readings were taken on the left and right arms of each subject in a sitting position after a 5-min rest between readings, and systolic and diastolic BPs were determined as their average. Height and body weight were measured to the nearest 0.1 cm or 0.1 kg, from which the BMI (kg/m^2^) was calculated as the weight in kilograms divided by the height in square meters. Blood samples were collected after a minimum 8-h fast for biochemical measurements. Fasting levels of blood glucose (mg/dL), total cholesterol (TC, mg/dL), HDL cholesterol (mg/dL), and TG (mg/dL) were measured using an automatic analyzer (ADVIA 1650 and 1680; Siemens, Tarrytown, NY, USA). LDL cholesterol was calculated using the Friedewald equation as follows: TC (mg/dL)−HDL cholesterol (mg/dL)−(TG (mg/dL))/5 in subjects with TG of <400 mg/dL [30].

### 2.4. Genotyping

DNA preparation and genotyping were performed as previously described for KoGES [29]. Briefly, DNA was isolated from peripheral blood leukocytes and genotyped using the Affymetrix Genome-Wide Human SNP Array 5.0 (Affymetrix, Inc., Santa Clara, CA, USA). For accuracy of genotyping, Bayesian robust linear modeling by the Mahalanobis distance genotyping algorithm was used. In KoGES-ASAS, a total of 352,228 SNPs became available after excluding SNPs with a high missing genotype call rate (>0.05) and/or low minor allele frequency (<0.01), those not in Hardy–Weinberg equilibrium (*p* < 1 × 10^−6^), and those with sex mismatch. After excluding 48,003 additional SNPs not in Hardy–Weinberg equilibrium at a significance of *p* < 1 × 10^−5^ through the EIGENSTRAT software package, the remaining 304,225 SNPs were subjected to further analyses. Among the 9 SNPs at *APOC3* previously known to be associated with circulating TG levels [15,31], in the present study, the genotypes of four SNPs at *APOC3* (T-455C, C-482T, C1100T, and SstI) available in the KoGES database were selected for testing the associations with cardiometabolic traits.

### 2.5. Definition of Incident HTN

Incident HTN was self-reported on the basis of biennial questionnaires. Subjects were considered to be positive for HTN if they were currently taking an anti-hypertensive medicine or undergoing treatment for HTN, had a previous diagnosis of HTN, or had systolic or diastolic BP >140 or >90 mmHg, respectively.

### 2.6. Statistical Analyses

The data are presented as means ± standard errors for continuous factors, and as counts and percentages for categorical factors. Mean differences between participants in general characteristics and biochemical parameters according to HTN were evaluated using Student’s *t*-test and chi-squared test for continuous and categorical variables, respectively. A generalized regression model was used to investigate linear trends in the biochemical markers after adjusting for age, gender, and residence (Ansan vs. Ansung). The relationships between the *APOC3* gene polymorphisms and cardiometabolic risk factors were examined using a linear regression model with an additive scale. To estimate the genetic effect of *APOC3* on HTN risk, Cox regression models with time-since-enrollment as a time-scale were used, including incident events occurring during follow-up. Cox proportional hazards models were constructed after adjusting for potential confounders (i.e., age, gender, area, total energy intake, physical activity, education level, smoking status, alcohol use, and BMI at baseline). In addition, we conducted analyses stratified by potential risk factors, including demographic and lifestyle measures. The results are presented as estimated hazard ratio (HR), and/or regression coefficient (β) with the 95% confidence interval (CI). All statistical analyses were performed using Stata SE 13.0 (StataCorp., College Station, TX, USA).

## 3. Results

### 3.1. Characteristics of Study Participants

The risk allele frequencies *APOC3* gene polymorphisms (C allele of T-455C, T allele of C-482T, C allele of C1100T, and G allele of SstI) were 0.469, 0.463, 0.403, and 0.324 respectively. General information and baseline characteristics of the study population are presented in Table 1. The mean age of total population was 50.0 ± 0.2 years, and the proportion of the male was 45.8%; 58.2% of participants lived in urban, Ansan. The average of METs and BMI were 18.5 ± 0.2 h/day and 24.06 ± 0.04 kg/m^2^, respectively. At baseline, the level of systolic and diastolic BPs, TG, and HDL cholesterol reached 112.3 mmHg, 74.8 mmHg, 143.0 mg/dL, and 45.3 mg/dL. Table 2 shows biochemical parameters of the population according to *APOC3* genotype. There were significant associations of all *APOC3* SNPs with TG levels. Subjects with the minor alleles of T-455C, C-482T, and SstI were more likely to have a higher TG level (β = 0.011, *p* = 0.009; β = 0.011, *p* = 0.011; and β = 0.017, *p* < 0.001, respectively), whereas participants having the C allele of the C1100T SNP had a significantly lower TG level and a higher HDL cholesterol level (β = −0.016, *p* < 0.001; and β = 0.515, *p* = 0.014).

### 3.2. Associations of APOC3 Gene Polymorphism with HTN Risk According to Risk Factors

**Figure 1:**
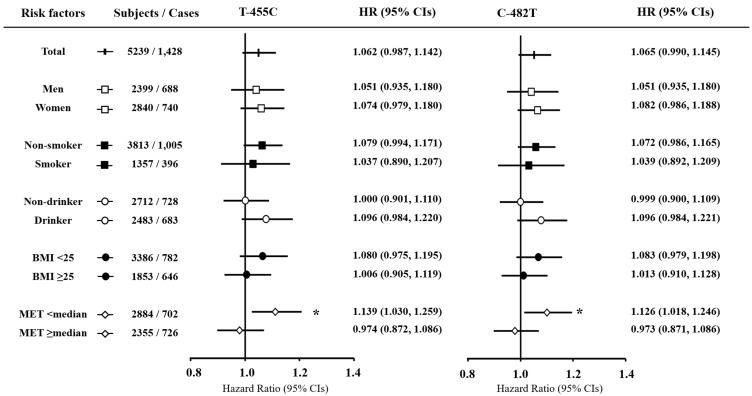

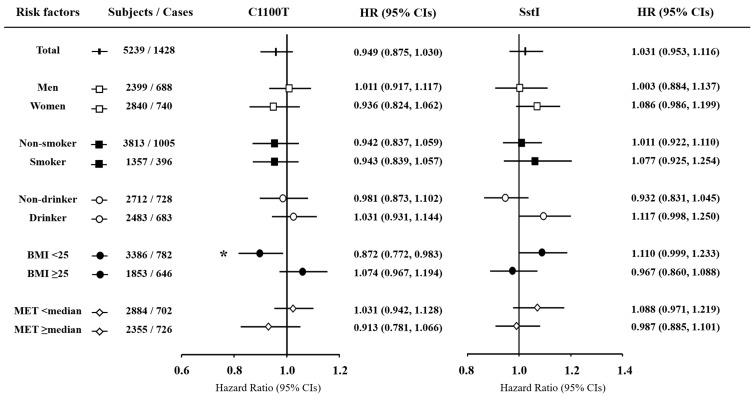
Associations of *APOC3* polymorphism with hypertension risk according to candidate lifestyle-related factors. BMI, body mass index; HR, hazard ratio; MET, metabolic equivalent. The median level of MET in the total population is 13.2 (h/day). Tested by Cox regression analyses adjusted for age, gender, area, total energy intake, physical activity, education level, smoking status, alcohol use, and BMI. Some variables included missing values. * Significant *p* value (*p* < 0.05).

**Figure 2:**
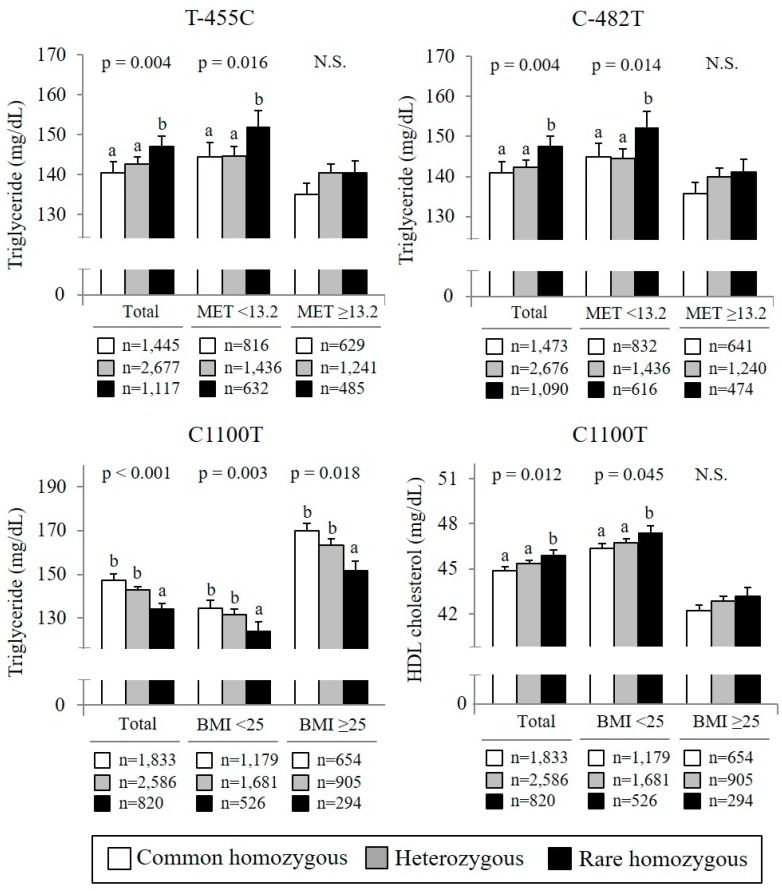
Association of *APOC3* genotype with triglyceride and HDL cholesterol by stratification for physical activity or BMI level. BMI, body mass index; HDL cholesterol, high-density-lipoprotein cholesterol; N.S., not significant; MET, metabolic equivalent. The median level of METs in the total population is 13.2 (h/day). Significance was tested using generalized linear model with Bonferroni’s multiple comparisons after adjusting for age, gender, area, total energy intake, physical activity, education level, smoking status, alcohol use, and BMI. Some variables included missing values. Circulating triglyceride was tested after log transformation. ^a,b^ The same lower-case letter means non-significant difference among groups.

After a median follow up of 9.8 years, 1428 participants had a diagnosis of HTN. Cox proportional regression analysis showed that none of the four tested SNPs (T-455C, C-482T, C1100T, and SstI) was associated with incident HTN in total subjects. We also performed the stratification analyses by dichotomizing the demographic factors, including gender, smoking and drinking status (current or nonsmoker/drinker), BMI (normal: BMI < 25 kg/m^2^ or obesity: BMI ≥ 25 kg/m^2^), and physical activity (METs <median or METs ≥median; the median of METs level in the total population was 13.2 h/day) (Figure 1). Based on the stratification analysis according to the level of METs, the C allele of T-455C and the T allele of C-482T were significantly related to HTN incidence in subjects with METs below the median (HR: 1.14, 95% CI: 1.03–1.26; HR: 1.13, 95% CI: 1.02–1.25). Correspondingly, a high level of physical activity attenuated HTN risk (Figure 1; HR: 1.06, 95% CI: 0.95–1.15). In the multivariate analysis, we assessed the discrepancy in the associations of APOC3 C1100T genotype with development of HTN, stratified by BMI levels. From the stratification analysis, among the subjects with BMI < 25 kg/m^2^, the TG-decreasing allele of C1100T was associated with a significantly lower HTN risk (Figure 1; HR: 0.87, 95% CI: 0.77–0.98). However, no significant result was observed among subjects with higher BMI. With regard to the associations with circulating levels of TG and HDL cholesterol, the C allele in T-455C and the T allele in C-482T were significantly related to higher levels of TG only in subjects with a low level of physical activity; however, in participants with a high level of physical activity, these relationships disappeared (Figure 2). In addition, among the subjects with BMI < 25 kg/m^2^, the TG-decreasing allele of C1100T was associated with a higher level of blood HDL cholesterol, whereas this was not observed among those with higher BMI (Figure 2).

## 4. Discussion

In the present study, the associations of four common genetic variants distributed across the *APOC3* gene region (T-455C, C-482T, SstI, and C1100T) with blood metabolic parameters and BP were tested in a community-based cohort from the Korean population. T-455C and C-482T are reported to confer resistance to insulin-mediated downregulation of *APOC3* gene transcription and induce elevated TG and HDL cholesterol levels and increased CVD risk [32]. In some studies, the C1100T and C3238G gene variants were associated with plasma apoC3 and TG concentrations [24,33], but other studies failed to replicate these associations [34,35]. We observed here that the minor alleles of T-455C and C-482T were significantly associated with a higher circulating TG level after adjusting for covariates, whereas the C allele of C1100T was associated with lower circulating TG and higher HDL cholesterol. Moreover, the minor allele of SstI was associated with a higher circulating TG level. Although the physiological mechanisms by which these genetic variations lead to alterations in TG metabolism remain unknown, the observed associations with circulating levels of TG and HDL cholesterol raise the question of whether lifelong exposure to either a beneficial or an atherogenic blood lipid profile due to genetic factors could be associated with future HTN development. In this study of a large prospective cohort that was initially free of HTN at baseline, Cox proportional regression analysis revealed that none of the tested genetic variants at *APOC3* was associated with incident HTN after a median 9.8-year follow-up. Although several previous studies reported associations of *APOC3* gene polymorphisms with the development of type 2 diabetes mellitus [36] and coronary artery disease [17,37], limited information is available as to whether genetic variations in *APOC3* predict HTN development. It was reported that *APOC3* 3238C/G SNP is associated with susceptibility to essential HTN in Egyptians [38]. In that study, it was speculated that the association of the variant with HTN was in part explained by its effect on blood levels of oxidized LDL [38]. A cross-sectional study of multi-ethnic populations also demonstrated that the -455C allele of *APOC3* was associated with high BP [39]. The discrepancy of this result with ours could be explained by ethnic differences in allele frequency [36], or other characteristics of the study participants, i.e., Koreans, as well as an insufficient length of follow-up. On the other hand, HTN is a complex disease in which there are strong interactions between genetic susceptibility and environmental factors. It has been shown that the relationship of *APOC3* genetic variants with metabolic syndrome risk is modified by diet [40]. It was also reported that variants in *APOC3* promoters increased type 2 diabetes risk only in lean populations, not in overweight individuals [41]. On the other hand, a previous study showing the effect of APOC3 mutations on the risk of ischemic vascular disease reported that there were no interactions between APOC3 genetic variants and some potential risk factors including BMI, physical inactivity, and HTN, which can be partly accounted for by the lack of statistical power [42]. In the present study, we next carried out the stratification analyses by certain lifestyle-related factors. Of interest is our finding that the significant association between two *APOC3* gene polymorphism (T-455C and C-482T) and incident HTN risk was only observed in participants with low physical activity. In addition, C1100T polymorphism was correlated with future HTN development among subjects with BMI < 25 kg/m^2^, while such an effect was not shown in obese participants. Regular physical activity is known to be associated with decreased circulating levels of TG [43] and increased levels of HDL cholesterol [44]. In addition, the effects of exercise on BP and incident HTN are well documented [45]. Our results demonstrated that the risk allele of the T-455C variant on the *APOC3* promoter might confer an increased HTN risk in individuals with decreased physical activity. Presumably, regular physical activity would blunt the genotype effects of these risk variants on circulating TG and future HTN development. The underlying mechanism behind the beneficial effect of the physical activity on *APOC3* genetic variants determining incident HTN requires further study. Endothelial dysfunction, arterial stiffness, and insulin resistance are likely to play important roles in this pathway [5,9,46]. It is generally accepted that endothelial dysfunction is integral not only in the atherosclerotic process, thrombosis, and insulin resistance, but also in the hypertensive process [5]. TG-rich lipoproteins have been shown to be toxic to endothelial cells [46]; therefore, it is possible that long-term exposure to endothelial dysfunction due to atherosclerotic dyslipidemia may lead to increased peripheral vascular resistance, and thus, to arterial HTN [5]. In addition, dyslipidemia has been shown to cause HTN by increasing arterial stiffness and decreasing arterial compliance of the carotid artery [46]. This lipoprotein lipase-mediated TG mechanism is further supported by previous results showing that physical activity at the population level is associated with improved endothelium [47], arterial stiffness [48], and insulin resistance [49]. Apart from the effects of *APOC3* on TG-rich lipoprotein metabolism, other pro-atherogenic and vascular effects of *APOC3* have been suggested [50], highlighting *APOC3* management as a therapeutic measure for cardiovascular risk reduction. Also, in the present study, we found that carriers of the C allele of C1100T associated with decreased TG and increased HDL cholesterol had a significantly lower HTN risk only when they were of normal weight. It can be speculated that in states of adequate insulin signaling, this SNP contributes to a healthy lipoprotein phenotype manifested by low TG and high HDL cholesterol, whereas in obesity, which is generally accompanied by insulin resistance, the SNPs became irrelevant for the regulation of *APOC3* expression, and consequently, their effects on TG metabolism may have been absent.

The strengths of our study include the large community-based sample of men and women, and the adjustment for potential confounders. In addition, the use of a relatively long follow-up allowed observation over time of the impact of *APOC3* gene variants on incident HTN. The use of self-reported outcomes could be considered a potential limitation, but self-reported HTN has been shown to be a valid and reliable measure [51,52]. Additionally, the lack of blood APOC3 measurement further limits the evaluation of the functional association of these variants with HTN development. Also, caution should be exercised in relation to the generalization of our findings, as this cohort was collected in only two communities for the subjects aged 40–69 years; in other words, they are not representative of the general Korean population.

## 5. Conclusions

In conclusion, we showed that the genotype effects of *APOC3* on HTN risk were evident in lean carriers of C allele of C1100T or in physically-inactive participants having the risk alleles of T-455C and C-482T in a prospective study with large Korean population. Also, we confirmed previous observations that common genetic variants at *APOC3* are associated with circulating levels of TG and HDL cholesterol. Exploring genetic susceptibility is pivotal for the personalized prevention of HTN. Our results confirm and extend current understanding of the contribution of *APOC3* gene polymorphism to the blood lipid profile and HTN risk in the Korean population.

## Figures and Tables

**Table 1:** General characteristics of the population at baseline.

	Total (*n* = 5239)
Age, years	50.0 (49.8, 50.3)
Male, % (n)	45.8 (2399)
Area, % (n)	
Ansung, urban	41.8 (2192)
Ansan, rural	58.2 (3047)
Education level, % (n)	
≤Elementary school	26.8 (1392)
≤Middle school	23.8 (1236)
≤High school	35.1 (1825)
≥University	14.3 (744)
Metabolic equivalents, h/day	18.5 (18.3, 18.7)
Current smoking, % (n)	26.3 (1357)
Current drinking, % (n)	47.8 (2483)
Body mass index, kg/m^2^	24.06 (24.02, 24.10)
Systolic BP, mmHg	112.3 (112.0, 112.6)
Diastolic BP, mmHg	74.8 (74.6, 75.0)
Biochemical markers	
Triglyceride, mg/dL	143.0 (141.0, 145.0)
Total cholesterol, mg/dL	187.4 (186.5, 188.3)
HDL cholesterol, mg/dL	45.3 (45.0, 45.6)
LDL cholesterol, mg/dL	114.0 (113.2, 114.8)
Fasting glucose, mg/dL	82.4 (82.2, 82.6)
Genotype **^a^**	
T-455C (TT/TC/CC, %)	27.6/51.1/21.3
C-482T (CC/CT/TT, %)	28.1/51.1/20.8
C1100T (TT/TC/CC, %)	35.0/49.4/15.7
SstI (CC/CG/GG, %)	45.1/44.9/10.0

BP, blood pressure; C, cytosine; G, guanine; HDL cholesterol, high-density-lipoprotein cholesterol; LDL cholesterol, low-density-lipoprotein cholesterol; T, thymine. Data are presented as means with ranges or the percentages for continuous and categorical variables. Some variables included missing values. ^a^ The genotype of each SNP was presented as common homozygous, heterozygous, and rare homozygous.

**Table 2:** Biochemical parameters of the population according to *APOC3* genotype.

Genotype ^a^	Triglyceride (mg/dL) ^b^	Total Cholesterol (mg/dL)	HDL Cholesterol (mg/dL)	LDL Cholesterol (mg/dL)	Fasting Glucose (mg/dL)
T-455C					
T/T (*n* = 1445)	140.9 (139.0, 142.8) ^c^	187.4 (186.5, 188.3)	45.3 (45.0, 45.6)	114.2 (113.4, 115.0)	82.5 (82.3, 82.7)
T/C (*n* = 2677)	142.4 (140.9, 143.9) ^cd^	187.3 (186.6, 188.0)	45.4 (45.2, 45.6)	113.9 (113.3, 114.5)	82.3 (82.1, 82.5)
C/C (*n* = 1117)	147.5 (145.2, 149.8) ^d^	187.8 (186.8, 188.8)	44.9 (44.6, 45.2)	114.1 (113.1, 115.1)	82.4 (82.2, 82.6)
*p* value	0.009	0.816	0.350	0.823	0.590
C-482T					
C/C (*n* = 1473)	140.4 (138.4, 142.4) ^c^	187.3 (186.4, 188.2)	45.3 (45.0, 45.6)	114.2 (113.4, 115.0)	82.5 (82.3, 82.7)
C/T (*n* = 2676)	142.7 (141.2, 144.2) ^cd^	187.5 (186.8, 188.2)	45.4 (45.2, 45.6)	113.9 (113.3, 114.5)	82.3 (82.1, 82.5)
T/T (*n* = 1090)	147.0 (144.7, 149.3) ^d^	187.6 (186.6, 188.6)	44.9 (44.6, 45.2)	114.0 (113.1, 114.9)	82.3 (82.1, 82.5)
*p* value	0.011	0.797	0.329	0.897	0.569
C1100T					
T/T (*n* = 1833)	147.2 (145.4, 149) ^d^	187.6 (186.8, 188.4)	44.9 (44.7, 45.1)	113.9 (113.2, 114.6)	82.4 (82.2, 82.6)
T/C (*n* = 2586)	142.8 (141.3, 144.3) ^d^	187.3 (186.6, 188.0)	45.4 (45.2, 45.6)	113.9 (113.3, 114.5)	82.3 (82.1, 82.5)
C/C (*n* = 820)	134.2 (131.8, 136.6) ^c^	187.2 (186.0, 188.4)	45.9 (45.5, 46.3)	114.7 (113.6, 115.8)	82.4 (82.1, 82.7)
*p* value	<0.001	0.744	0.014	0.620	0.884
SstI					
C/C (*n* = 2365)	139.4 (137.9, 140.9) ^c^	187.2 (186.5, 187.9)	45.5 (45.3, 45.7)	114.2 (113.6, 114.8)	82.3 (82.1, 82.5)
C/G (*n* = 2352)	143.9 (142.4, 145.4) ^c^	187.4 (186.7, 188.1)	45.3 (45.1, 45.5)	113.9 (113.3, 114.5)	82.4 (82.2, 82.6)
G/G (*n* = 522)	155.4 (151.7, 159.1) ^d^	188.2 (186.7, 189.7)	44.5 (44.1, 44.9)	113.8 (112.4, 115.2)	82.7 (82.3, 83.1)
*p* value	<0.001	0.590	0.052	0.697	0.302

C, cytosine; G, guanine; HDL cholesterol, high-density-lipoprotein cholesterol; LDL cholesterol, low-density-lipoprotein cholesterol; T, thymine. Data are presented as means with ranges for clinical parameters. Differences in the genotype were determined using Student’s analysis of variance (ANOVA). Significance was tested using generalized linear model. Some variables included missing values. ^a^ The genotype of each SNP was presented as common homozygous, heterozygous, and rare homozygous. ^b^ Circulation triglyceride was tested after log transformation. ^c,d^ The same lower-case letter means non-significant difference among groups.

## References

[1] He J., Gu D., Chen J., Wu X., Kelly T.N., Huang J.F., Chen J.C., Chen C.S., Bazzano L.A., Reynolds K. (2009). Premature deaths attributable to blood pressure in China: A prospective cohort study. Lancet.

[2] Korea Centers for Disease Control and Prevention (2017). Korea Health Statistics: Korea National Health and Nutrition Examination Survey (KNHANES VII).

[3] Xi B., Cheng H., Shen Y., Zhao X., Hou D., Wang X., Mi J. (2012). Physical activity modifies the associations between genetic variants and hypertension in the Chinese children. Atherosclerosis.

[4] Freiberg J.J., Tybjaerg-Hansen A., Jensen J.S., Nordestgaard B.G. (2008). Nonfasting triglycerides and risk of ischemic stroke in the general population. JAMA.

[5] Laaksonen D.E., Niskanen L., Nyyssonen K., Lakka T.A., Laukkanen J.A., Salonen J.T. (2008). Dyslipidaemia as a predictor of hypertension in middle-aged men. Eur. Heart. J..

[6] Kasahara A., Adachi H., Hirai Y., Enomoto M., Fukami A., Yoshikawa K., Esaki E., Yokoi K., Ogata K., Tsukagawa E. (2013). High Level of Plasma Remnant-like Particle Cholesterol May Predispose to Development of Hypertension in Normotensive Subjects. Am. J. Hypertens..

[7] Skarn S.N., Flaa A., Kjeldsen S.E., Rostrup M., Brunborg C., Reims H.M., Fossum E., Hoieggen A., Aksnes T.A. (2015). Family history of hypertension and serum triglycerides predict future insulin sensitivity: A 17-year follow-up study of young men. J. Hypertens..

[8] Shimbo D., Muntner P., Mann D., Viera A.J., Homma S., Polak J.F., Barr R.G., Herrington D., Shea S. (2010). Endothelial Dysfunction and the Risk of Hypertension: The Multi-Ethnic Study of Atherosclerosis. Hypertension.

[9] Urbina E.M., Srinivasan S.R., Kieltyka R.L., Tang R., Bond M.G., Chen W., Berenson G.S. (2004). Correlates of carotid artery stiffness in young adults: The Bogalusa heart study. Atherosclerosis.

[10] Franco O.H., Massaro J.M., Civil J., Cobain M.R., O’Malley B., D’Agostino R.B. (2009). Trajectories of Entering the Metabolic Syndrome: The Framingham Heart Study. Circulation.

[11] Norata G.D., Tsimikas S., Pirillo A., Catapano A.L. (2015). Apolipoprotein C-III: From Pathophysiology to Pharmacology. Trends Pharmacol. Sci..

[12] Kawakami A., Aikawa M., Alcaide P., Luscinskas F.W., Libby P., Sacks F.M. (2006). Apolipoprotein CIII induces expression of vascular cell adhesion molecule-1 in vascular endothelial cells and increases adhesion of monocytic cells. Circulation.

[13] Tao Y., Xiong Y.S., Wang H.M., Chu S.P., Zhong R.Q., Wang J.X., Wang G.H., Ren X.M., Yu J. (2016). APOC3 induces endothelial dysfunction through TNF-alpha and JAM-1. Lipids Health Dis..

[14] Puppala J., Bhrugumalla S., Kumar A., Siddapuram S.P., Viswa P.D., Kondawar M., Akka J., Munshi A. (2014). Apolipoprotein C3 gene polymorphisms in Southern Indian patients with nonalcoholic fatty liver disease. Indian J. Gastroenterol..

[15] Crosby J., Peloso G.M., Auer P.L., Crosslin D.R., Stitziel N.O., Lange L.A., Lu Y., Tang Z.Z., Zhang H., TG and HDL Working Group of the Exome Sequencing Project, National Heart, Lung, and Blood Institute (2014). Loss-of-function mutations in APOC3, triglycerides, and coronary disease. N. Engl. J. Med..

[16] Surguchov A.P., Page G.P., Smith L., Patsch W., Boerwinkle E. (1996). Polymorphic markers in apolipoprotein C-III gene flanking regions and hypertriglyceridemia. Arterioscler. Thromb. Vasc. Biol..

[17] Lin B., Huang Y.W., Zhang M.Y., Wang J., Wu Y.H. (2014). Association between apolipoprotein C3 Sst I, T-455C, C-482T and C1100T polymorphisms and risk of coronary heart disease. BMJ Open.

[18] Petersen K.F., Dufour S., Hariri A., Nelson-Williams C., Foo J.N., Zhang X.M., Dziura J., Lifton R.P., Shulman G.I. (2010). Apolipoprotein C3 Gene Variants in Nonalcoholic Fatty Liver Disease. N. Engl. J. Med..

[19] Corella D., Guillen M., Saiz C., Portoles O., Sabater A., Folch J., Ordovas J.M. (2002). Associations of LPL and APOC3 gene polymorphisms on plasma lipids in a Mediterranean population: Interaction with tobacco smoking and the APOE locus. J. Lipid Res..

[20] Kozlitina J., Boerwinkle E., Cohen J.C., Hobbs H.H. (2011). Dissociation Between APOC3 Variants, Hepatic Triglyceride Content and Insulin Resistance. Hepatology.

[21] Kee F., Amouyel P., Fumeron F., Arveiler D., Cambou J.P., Evans A., Cambien F., Fruchart J.C., Ducimetiere P., Dallongeville J. (1999). Lack of association between genetic variations of apo A-I-C-III-A-IV gene cluster and myocardial infarction in a sample of European male: ECTIM study. Atherosclerosis.

[22] Niu T.H., Jiang M., Xin Y.N., Jiang X.J., Lin Z.H., Xuan S.Y. (2014). Lack of association between apolipoprotein C3 gene polymorphisms and risk of nonalcoholic fatty liver disease in a Chinese Han population. World J. Gastroenterol..

[23] Zhang H., Chen L., Xin Y., Lou Y., Liu Y., Xuan S. (2014). Apolipoprotein c3 gene polymorphisms are not a risk factor for developing non-alcoholic Fatty liver disease: A meta-analysis. Hepat. Mon..

[24] Ribalta J., LaVille A.E., Vallve J.C., Humphries S., Turner P.R., Masana L. (1997). A variation in the apolipoprotein C-III gene is associated with an increased number of circulating VLDL and IDL particles in familiar combined hyperlipidemia. J. Lipid Res..

[25] Tilly P., Sass C., Vincent-Viry M., Aguillon D., Siest G., Visvikis S. (2003). Biological and genetic determinants of serum apoC-III concentration: Reference limits from the Stanislas Cohort. J. Lipid Res..

[26] Kim Y., Han B.G., Grp K. (2017). Cohort Profile: The Korean Genome and Epidemiology Study (KoGES) Consortium (vol 46, pg e20, 2016). Int. J. Epidemiol..

[27] Ahn Y., Kwon E., Shim J.E., Park M.K., Joo Y., Kimm K., Park C., Kim D.H. (2007). Validation and reproducibility of food frequency questionnaire for Korean genome epidemiologic study. Eur. J. Clin. Nutr..

[28] Ainsworth B.E., Haskell W.L., Whitt M.C., Irwin M.L., Swartz A.M., Strath S.J., O’Brien W.L., Bassett D.R., Schmitz K.H., Emplaincourt P.O. (2000). Compendium of Physical Activities: An update of activity codes and MET intensities. Med. Sci. Sport Exerc..

[29] Cho Y.S., Go M.J., Kim Y.J., Heo J.Y., Oh J.H., Ban H.J., Yoon D., Lee M.H., Kim D.J., Park M. (2009). A large-scale genome-wide association study of Asian populations uncovers genetic factors influencing eight quantitative traits. Nat. Genet..

[30] Friedewald W.T., Levy R.I., Fredrickson D.S. (1972). Estimation of the concentration of low-density lipoprotein cholesterol in plasma, without use of the preparative ultracentrifuge. Clin. Chem..

[31] Zhang R.N., Zheng R.D., Mi Y.Q., Zhou D., Shen F., Chen G.Y., Zhu C.Y., Pan Q., Fan J.G. (2016). APOC3 rs2070666 Is Associated with the Hepatic Steatosis Independently of PNPLA3 rs738409 in Chinese Han Patients with Nonalcoholic Fatty Liver Diseases. Dig. Dis. Sci..

[32] Peter A., Kantartzis K., Machicao F., Machann J., Wagner S., Templin S., Konigsrainer I., Konigsrainer A., Schick F., Fritsche A. (2012). Visceral obesity modulates the impact of apolipoprotein C3 gene variants on liver fat content. Int. J. Obes..

[33] Hosseini-Esfahani F., Mirmiran P., Daneshpour M.S., Mottaghi A., Azizi F. (2017). The Effect of Interactions of Single Nucleotide Polymorphisms of APOA1/APOC3 with Food Group Intakes on the Risk of Metabolic Syndrome. Avicenna J. Med. Biotechnol..

[34] Olivieri O., Stranieri C., Bassi A., Zaia B., Girelli D., Pizzolo F., Trabetti E., Cheng S., Grow M.A., Pignatti P.F. (2002). ApoC-III gene polymorphisms and risk of coronary artery disease. J. Lipid Res..

[35] Russo G.T., Meigs J.B., Cupples L.A., Demissie S., Otvos J.D., Wilson P.W., Lahoz C., Cucinotta D., Couture P., Mallory T. (2001). Association of the Sst-I polymorphism at the APOC3 gene locus with variations in lipid levels, lipoprotein subclass profiles and coronary heart disease risk: The Framingham offspring study. Atherosclerosis.

[36] Onat A., Erginel-Unaltuna N., Coban N., Cicek G., Yuksel H. (2011). APOC3 -482C > T polymorphism, circulating apolipoprotein C-III and smoking: Interrelation and roles in predicting type-2 diabetes and coronary disease. Clin. Biochem..

[37] Liu S., Song Y., Hu F.B., Niu T., Ma J., Gaziano M., Stampfer M.J. (2004). A prospective study of the APOA1 XmnI and APOC3 SstI polymorphisms in the APOA1/C3/A4 gene cluster and risk of incident myocardial infarction in men. Atherosclerosis.

[38] Ghattas M., Badawy H., Mesbah N., Abo-Elmatty D. (2013). Apolipoprotein CIII3238C/G gene polymorphism influences oxidized low-density lipoprotein with a risk of essential hypertension. J. Biochem. Pharmacol. Res..

[39] Pollex R.L., Ban M.R., Young T.K., Bjerregaard P., Anand S.S., Yusuf S., Zinman B., Harris S.B., Hanley A.J.G., Connelly P.W. (2007). Association between the −455T > C promoter polymorphism of the APOC3 gene and the metabolic syndrome in a multi-ethnic sample. BMC Med. Genet..

[40] Hosseini-Esfahani F., Mirmiran P., Daneshpour M.S., Mehrabi Y., Hedayati M., Soheilian-Khorzoghi M., Azizi F. (2015). Dietary patterns interact with APOA1/APOC3 polymorphisms to alter the risk of the metabolic syndrome: The Tehran Lipid and Glucose Study. Br. J. Nutr..

[41] van Hoek M., van Herpt T.W., Dehghan A., Hofman A., Lieverse A.G., van Duijn C.M., Witteman J.C.M., Sijbrands E.J.G. (2011). Association of an APOC3 promoter variant with type 2 diabetes risk and need for insulin treatment in lean persons. Diabetologia.

[42] Jorgensen A.B., Frikke-Schmidt R., Nordestgaard B.G., Tybjaerg-Hansen A. (2014). Loss-of-function mutations in APOC3 and risk of ischemic vascular disease. N. Engl. J. Med..

[43] Mostafavi F., Ghofranipour F., Feizi A., Pirzadeh A. (2015). Improving Physical Activity and Metabolic Syndrome Indicators in Women: A Transtheoretical Model-Based Intervention. Int. J. Prev. Med..

[44] Alkahtani S., Elkilany A., Alhariri M. (2015). Association between sedentary and physical activity patterns and risk factors of metabolic syndrome in Saudi men: A cross-sectional study. BMC Public Health.

[45] Liu X.J., Zhang D.D., Liu Y., Sun X.Z., Han C.Y., Wang B.Y., Ren Y.C., Zhou J.M., Zhao Y., Shi Y.Y. (2017). Dose-Response Association Between Physical Activity and Incident Hypertension A Systematic Review and Meta-Analysis of Cohort Studies. Hypertension.

[46] O’Connell B.J., Genest J. (2001). High-density lipoproteins and endothelial function. Circulation.

[47] Bey L., Hamilton M.T. (2003). Suppression of skeletal muscle lipoprotein lipase activity during physical inactivity: A molecular reason to maintain daily low-intensity activity. J. Physiol. Lond..

[48] Augustine J., Tarzia B., Kasprowicz A., Heffernan K.S. (2014). Effect of a Single Bout of Resistance Exercise on Arterial Stiffness Following a High-Fat Meal. Int. J. Sports Med..

[49] Petibois C., Cassaigne A., Gin H., Deleris G. (2004). Lipid profile disorders induced by long-term cessation of physical activity in previously highly endurance-trained subjects. J. Clin. Endocr. Metab..

[50] Kawakami A., Aikawa M., Nitta N., Yoshida M., Libby P., Sacks F.M. (2007). Apolipoprotein CIII-induced THP-1 cell adhesion to endothelial cells involves pertussis toxin-sensitive G protein- and protein kinase C alpha-mediated nuclear factor-kappa B activation. Arterioscler. Thromb. Vasc. Biol..

[51] Paynter N.P., Sesso H.D., Conen D., Otvos J.D., Mora S. (2011). Lipoprotein Subclass Abnormalities and Incident Hypertension in Initially Healthy Women. Clin. Chem..

[52] Colditz G.A., Martin P., Stampfer M.J., Willett W.C., Sampson L., Rosner B., Hennekens C.H., Speizer F.E. (1986). Validation of Questionnaire Information on Risk-Factors and Disease Outcomes in a Prospective Cohort Study of Women. Am. J. Epidemiol..

